# In Situ Sustained Macrophage-Targeted Nanomicelle–Hydrogel Microspheres for Inhibiting Osteoarthritis

**DOI:** 10.34133/research.0131

**Published:** 2023-05-02

**Authors:** XiaoXiao Li, Xingchen Li, Jielai Yang, Yawei Du, Liang Chen, Gang Zhao, Tingjun Ye, Yuan Zhu, Xiangyang Xu, Lianfu Deng, Wenguo Cui

**Affiliations:** Department of Orthopaedics, Shanghai Key Laboratory for Prevention and Treatment of Bone and Joint Diseases, Shanghai Institute of Traumatology and Orthopaedics, Ruijin Hospital, Shanghai Jiao Tong University School of Medicine, 197 Ruijin 2nd Road, Shanghai 200025, P. R. China.

## Abstract

There are still challenges in applying drug nanocarriers for in situ sustained macrophage targeting and regulation, due to the rapid clearance of nanocarriers and burst drug release in vivo. Herein, a nanomicelle–hydrogel microsphere, characterized by its macrophage-targeted nanosized secondary structure that allows it to accurately bind to M1 macrophages through active endocytosis, is employed for in situ sustained macrophage targeting and regulation, and addresses the insufficient osteoarthritis therapeutic efficacy caused by rapid clearance of drug nanocarriers. The 3-dimensional structure of a microsphere can prevent the rapid escape and clearance of a nanomicelle, thus keeping it in joints, while the ligand-guided secondary structure can carry drugs to accurately target and enter M1 macrophages, and release drugs via the transition from hydrophobicity to hydrophilicity of nanomicelles under inflammatory stimulation inside the macrophages. The experiments show that the nanomicelle–hydrogel microsphere can in situ sustainably target and regulate M1 macrophages for more than 14 days in joints, and attenuate local “cytokine storm” by continuous M1 macrophage apoptosis promotion and polarization inhibition. This micro/nano-hydrogel system shows excellent ability to sustainably target and regulate macrophage, realizes the improvement of drug utilization and efficacy inside the macrophage, and thereby can be a potential platform for treating macrophage-related diseases.

## Introduction

Macrophages are one of the most important intrinsic immune cells that can respond to microenvironmental signals and differentiate to match the characteristics, phenotype, and function of the local environment by a dynamic and rapid process called macrophage polarization [1,2]. The polarization of macrophages is a dynamic process mediated by various intracellular small molecules and signaling pathways, which can be modulated in response to the local microenvironment [3–5]. In contrast, shifts of macrophage-related genes and cytokines are expected in the adaptation of the phenotype switch, which, in turn, affect the local microenvironment [6,7].

Depending on the stimulus, macrophages are usually polarized into 2 phenotypes: the pro-inflammatory M1 that secretes pro-inflammatory factors and the anti-inflammatory M2 that promotes tissue repair [8]. Macrophages are widely distributed throughout the body and participate in the pathogenesis and progression of various diseases [9]. For example, inflammation-related diseases, including osteoarthritis (OA), rheumatoid arthritis, and Crohn’s disease, often involve the overactivation of M1 macrophages [10]. Therefore, it would be meaningful to establish macrophage-targeted systems for intracellular delivery of therapeutic drugs with high efficiency and selectivity, achieving modulation of macrophages and the subsequent diseases they triggered.

Nanocarriers, such as polymeric nanomicelles, liposomes, and dendrimers, have been utilized for macrophage-targeted therapies due to their unique size and potential capabilities to enhance the targetability and stability of encapsulated drugs [11,12]. The targeting effects of nanocarriers involve active targeting and passive targeting based on the underlying targeting mechanisms. Passive targeting of nanoparticles is facilitated by the relatively small particle size, electrostatic interactions, and the hydrophobicity and hydrophilicity of nanocarriers [13–15]. Active targeting often involves active endocytosis that is mediated by the specific binding interactions between ligands modified on the nanocarrier surface and receptors (e.g., folate receptors) on the cell membrane [16,17]. Increased local therapeutic drug concentration and reduced drug uptake by non-targeted cells are among the advantages of the active targeting strategy [18,19]. However, challenges remain for active/passive targeting. Once delivered locally, unintentional elimination of nanocarriers by body fluid exchange seems inevitable in vivo, impeding the long-active/passive in situ targetability and therapeutic effects [20,21]. Hydrogel microspheres have emerged as novel and versatile drug delivery systems with excellent biocompatibility and high modifiability [22–24]. It has been proven that hydrogel microspheres offered physical protection and controlled release of the encapsulated nanocarriers, prohibiting the rapid clearance caused by the body fluid exchange and maintaining the therapeutic drug concentration in situ [25–27]. Lin et al. [14] loaded the nanocarriers polyamidoamine with kartogenin, then the drug-loaded nanocarriers were further encapsulated in hyaluronic acid methacrylate. These hydrogel microspheres could release kartogenin for more than 21 days and considerably delayed OA progression. Therefore, it would be reasonable to combine hydrogel microspheres with targeted nanocarriers to construct a nanocarrier–hydrogel microsphere drug delivery system that could maintain the controlled release of nanocarriers and the therapeutic drug concentration in situ.

The innate immunity dominated by macrophages is the leading cause of the development and progression of early OA. According to the literatures, M1 macrophage overactivation and suppression of M2 macrophage polarization can result in imbalanced M1/M2 [6,28]. The activated M1 macrophages can release large amounts of chemokines and pro-inflammatory factors, including tumor necrosis factor-α (TNF-α), interleukin-1 (IL-1), and interleukin-6 (IL-6). Consequently, high concentrations of chemokines can further recruit inflammatory cells to the inflammatory zone, considerably escalating inflammatory cell infiltration and triggering a local “cytokine storm”, exacerbating the OA microenvironment [29,30]. To make things worse, activated M1 macrophages also generate reactive oxygen species (ROS), which can further facilitate M1 macrophage polarization, aggravating the inflammatory vicious cycle [31,32]. Therefore, it would be essential to develop an in situ macrophage-targeted system that could inhibit “cytokine storm” and delay OA progression by precisely delivering drugs to M1 macrophages with simultaneous M1 apoptosis promotive and M1 polarization inhibitive properties [33].

**Fig. 1.**
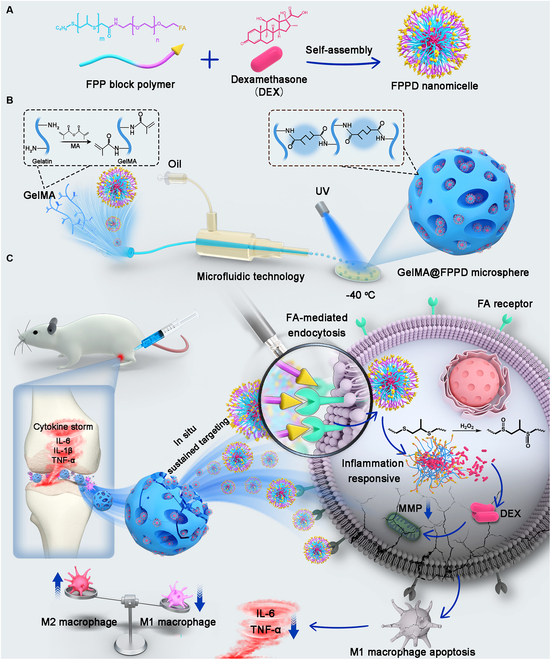
Schematic diagram of nanomicelle–hydrogel microspheres (GelMA@FPPD) that sustainably target and regulate M1 macrophage, inhibit “cytokine storm”, and alleviate OA. (A) Fabrication of FPPD nanomicelles. (B) Fabrication of FPPD nanomicelle-loaded hydrogel microspheres (GelMA@FPPD). (C) When delivered intra-articularly into the knee joint, GelMA@FPPD could inhibit “cytokine storm” and alleviate OA by in situ sustained M1 macrophage targeting, inflammation inhibition, and M1/M2 rebalancing.

Based on the characteristics of OA, the injectable nanomicelle–hydrogel microspheres with the in situ sustained macrophage targetability were constructed in this study via microfluidic technology and nanotechnology to alleviate OA progression, addressing the insufficient therapeutic efficacy caused by rapid clearance of drug nanocarriers (Fig. 1). Specifically, the dexamethasone (DEX)-loaded nanomicelles (FPPD), fabricated by the hydrophobic self-assembly process of folate acid–polyethylene glycol–polypropylene sulfide block polymers (FA–PEG–PPS), were used as secondary structures and entrapped into GelMA microspheres via microfluidic technology, thus establishing the M1 macrophage-targeted micro/nano drug delivery system (GelMA@FPPD). The hydrogel network of GelMA microspheres could not only provide excellent protection of FPPD against rapid clearance but also maintain the in situ controlled release of FPPD within the joints. Moreover, the sustainably released FPPD could target and enter M1 macrophages via folate acid receptor-mediated endocytosis, and then release DEX under inflammatory stimulation inside the macrophages via the transition from hydrophobicity to hydrophilicity of polypropylene sulfide groups. By sustainably targeting and promoting M1 macrophage apoptosis and inhibiting M1 polarization, GelMA@FPPD could downregulate inflammatory factor expressions, inhibit local “cytokine storm”, and alleviate cartilage destruction and OA progression. Overall, the novel nanomicelle–hydrogel microspheres that sustainably target macrophages exhibited great potential in the minimally invasive treatment of OA and other macrophage-mediated diseases.

## Results and Discussion

### Fabrication of macrophage-targeted nanomicelle–hydrogel microspheres

#### Fabrication of ROS-responsive macrophage-targeted nanomicelles (FPP)

**Fig. 2.**
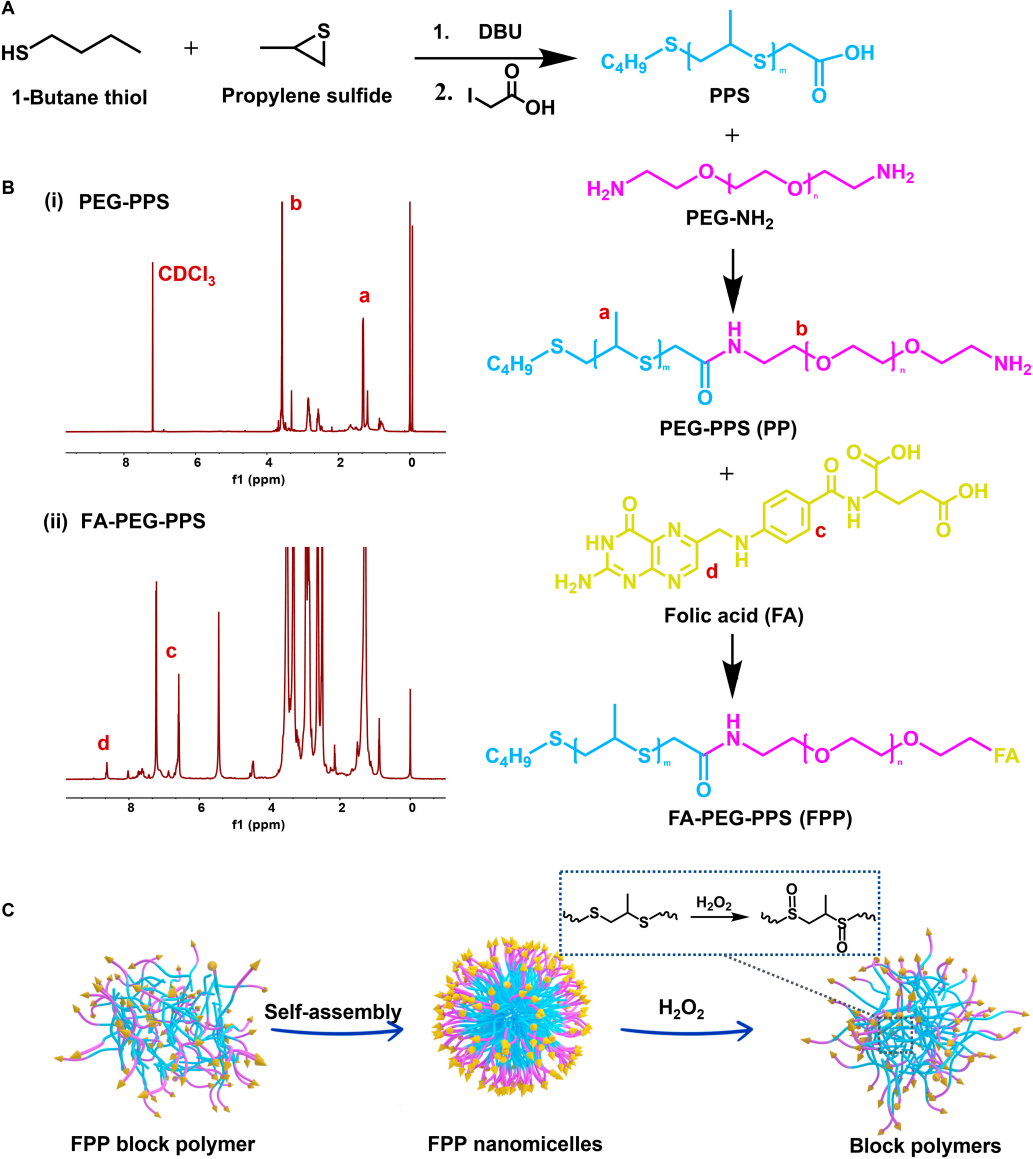
Fabrication of FPP nanomicelles. (A) Fabrication of PP and FPP block polymers. (B) ^1^H NMR spectra of PP and FPP block polymers. (C) Self-assembly and ROS-responsive dissociation of FPP nanomicelles under ROS oxidation stress. FPP nanomicelles were composed of hydrophilic shells (FA-PEG) and hydrophobic cores (thioether groups), which were able to oxidize to hydrophilic sulfoxide groups in response to ROS oxidation stress, triggering dissociation of nanomicelles.

Nanomicelles (5 to 200 nm) with hydrophobic cores and hydrophilic shells are formed by the self-assembly of polymers with amphiphilic chain segments in water due to the entropic hydrophobic effect [34,35]. Owing to their unique structure, nanomicelles can effectively enhance the solubility and stability of hydrophobic drugs by loading them via specific biochemical/physical interactions. According to the specific therapeutic demands, the amphiphilic polymeric structures in the micellar delivery system can be modified with various responsive properties, such as targetability and ROS responsiveness [36]. Therefore, according to the methods in previous studies [37,38], a PEG-PPS (PP) block polymer consisting of PEG as the hydrophilic segment and PPS as the hydrophobic segment was successfully prepared in this study (Fig. 2A). Then, the FA was grafted onto the PEG chain segment by using EDC/DMAP coupling chemistry to form FA–PEG–PPS block polymer (FPP). The successful synthesis of these block polymers was proved by the ^1^H-nuclear magnetic resonance (^1^H NMR) results (Fig. 2B).

**Fig. 3.**
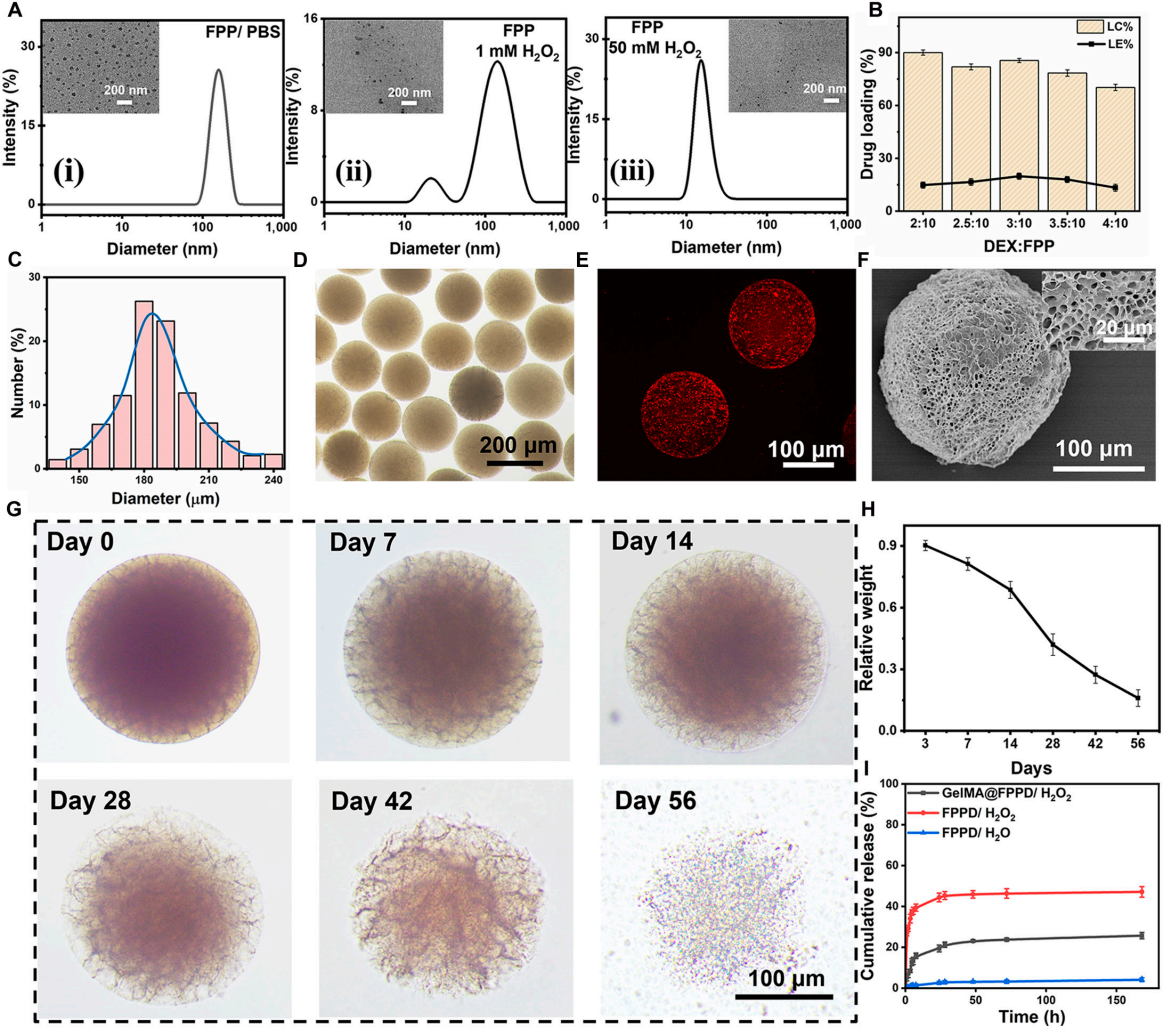
Physical characterization of nanomicelle–GelMA microspheres. (A) The hydrodynamic diameter and TEM images of FPP nanomicelles in PBS and H_2_O_2_. (B) Evaluation of the drug loading capacity (LC%) and loading efficacy (LE%) of DEX in FPP nanomicelles (*n* = 3). (C and D) The diameter distribution and bright-field microscopic image of nanomicelle–GelMA microspheres. (E) Confocal microscopic image demonstrated the distribution of FPP nanomicelles in GelMA microspheres. FPP nanomicelles were labeled with Nile Red with red fluorescence. (F) The porous structure of GelMA microspheres was clearly presented on the SEM image. (G and H) Morphological evaluation of GelMA microspheres under bright-field microscopy in vitro and the degradation curve (*n* = 3). (I) DEX drug release profiles in FPPD and GelMA@FPPD under H_2_O and 1 mM H_2_O_2_ (*n* = 3).

FPP nanomicelles with PPS as hydrophobic core and FA-PEG as hydrophilic shell were prepared by solvent evaporation with a concentration higher than the critical micelle concentration (Fig. 2C) [39]. In FPP nanomicelles, the PPS chain segments were reductive, and the hydrophobic thioether groups could be oxidized to hydrophilic sulfoxide groups under H_2_O_2_ conditions [40]. Therefore, the responsive dissociation of nanomicelles could be achieved by the transition between the hydrophobicity and hydrophilicity of PPS chain segments under oxidation stress. To explore the formation and dissociation of FPP nanomicelles, 0 mM, 1 mM, and 50 mM of H_2_O_2_ were added to the FPP nanomicelle solution. The particle size and morphology of FPP were evaluated by dynamic light scattering and transmission electron microscopy (TEM). The hydrodynamic diameter of FPP was about 150 nm in phosphate buffered saline (PBS) (Fig. 3A), and the TEM images showed that FPP was spherical in shape, which indicated that FPP can self-assemble into nanomicelles and maintain the micellar structure in PBS or H_2_O for a long time (Fig. S1). However, under different concentrations of H_2_O_2_, the hydrophobicity–hydrophilicity transition of PPS chain segments caused partial or complete dissociation of FPP nanomicelles with a hydrodynamic diameter about 20 nm, indicating the specific response of FPP nanomicelles to H_2_O_2_.

#### Loading of FPPD in GelMA microspheres and the sustained release of DEX

DEX is a hydrophobic glucocorticoid with anti-inflammatory properties, which changes the inflammatory microenvironment by inhibiting the accumulation of inflammatory cells (e.g., macrophages and leukocytes) and has been widely used in the treatment of OA [41,42]. In this study, DEX was loaded during the self-assembly process of FPP block polymers into nanomicelles in water to prepare FPPD. The FPP nanomicelles exhibited excellent encapsulation properties with an efficient drug loading capacity (LC%) of up to 90% for DEX (Fig. 3B). Subsequently, GelMA@FPPD with a hydrodynamic diameter of around 200 μm were prepared by mixing FPPD micelles with GelMA monomer as the aqueous phase in microfluidic technology. GelMA@FPPD demonstrated excellent monodispersity under bright-field microscopy (Fig. 3C and D). To verify the successful immobilization of FPP within the hydrogel microspheres, FPP was labeled with Nile Red. As shown in Fig. 3E, red fluorescence could be observed in GelMA@FPPD, while there was almost no red fluorescence in the surrounding solution, indicating that FPP nanomicelles were effectively immobilized within the GelMA microsphere network.

The porous structure and degradation properties of microspheres could not only avoid the rapid clearance of the small-molecule drugs or nanomicelles but also enable their sustained release to achieve in situ long-acting treatment [43,44]. Therefore, in this study, the degradation properties of GelMA microspheres were evaluated by bright-field microscopy since the GelMA backbone that is derived from collagen can be dissociated under the condition containing collagenase II [25]. Their porous structure was investigated by scanning electron microscopy (SEM). As shown in Fig. 3F, the freshly prepared GelMA microspheres showed a dense 3-dimensional network structure under SEM, and the bright-field microscopy validated their biodegradability over time (Fig. 3G and H), suggesting that the porous structure of GelMA microspheres enabled the controlled release of FPPD nanomicelles and DEX. The cumulative release rate of DEX from FPP nanomicelles and GelMA@FPPD under H_2_O/H_2_O_2_ was assessed by ultraviolet-visible spectroscopy. Limited releases of DEX were observed in FPPD under H_2_O, while under the oxidation stress of 1 mM H_2_O_2_, a sudden release of DEX occurred due to the dissociation of the micelle structure (Fig. 3I). For GelMA@FPPD, a moderate and controlled release profile of DEX was observed due to the dense network of GelMA microspheres that prevented the rapid diffusion of small molecules and nanomicelles.

### Sustained macrophage-targeted and ROS-scavenging properties of GelMA@FPPD

**Fig. 4.**
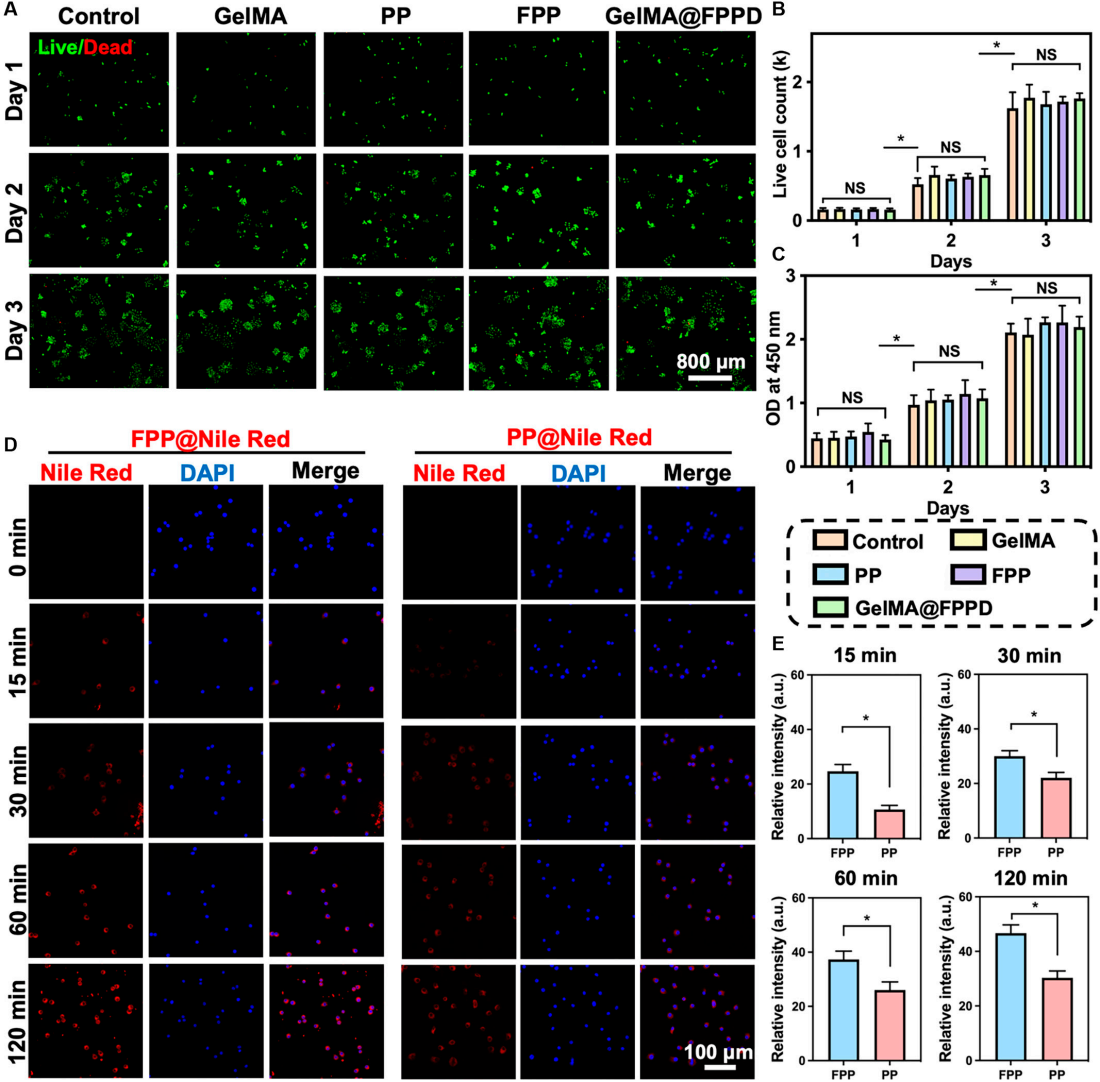
Biocompatibility of GelMA@FPPD and endocytosis of FPP nanomicelles by activated RAW 264.7 macrophages. (A) Representative live/dead staining images in different groups from day 1 to day 3. (B) Live cell counts from day 1 to day 3 in different groups. (C) Optical density (OD) values in CCK8 assays from day 1 to day 3 in different groups. (D) Representative confocal images demonstrated the endocytosis of FPP@Nile Red and PP@Nile Red by activated RAW 264.7 macrophages at designated time points. Nucleus: blue; Nile Red-labeled nanomicelles: red. (E) Intracellular relative fluorescence intensity at designated time points (*n* = 3, NS: no significance, * indicates *P* < 0.05).

According to previous studies, FA receptors are highly overexpressed on the cell membrane of M1 macrophages [17]. Therefore, the M1 macrophage-targeted drug delivery system could be achieved by harnessing the specific binding property of FA to its receptors[16,45,46]. Herein, the biocompatibility of GelMA@FPPD was firstly evaluated by live/dead staining and cell count kit-8 assay (CCK8). After 3 days of co-culture with GelMA@FPPD, a substantial increase in the number of Raw 264.7 macrophages was observed (Fig. 4A to C), indicating their considerable biocompatibility. Moreover, the hemolysis activity test also demonstrated that GelMA@FPPD had satisfactory hemocompatibility (Fig. S2). Subsequently, to verify the macrophage targeting capacity of FPP, Nile Red was encapsulated inside the nanomicelles and co-cultured with activated M1 macrophages. Intracellular red fluorescence intensity was evaluated by using confocal microscopy at specific time points (Fig. 4D and E). After drug addition, the fluorescence intensities for both groups increased with the increasing incubation time, and the average relative intracellular red fluorescence intensity was 46.7 ± 3.1 in the FPP group and 30.3 ± 2.5 in the PP group at 120 min, which might indicate that the modification of FA on the shell of FPP facilitated the FA-mediated endocytosis of macrophages [47].

**Fig. 5.**
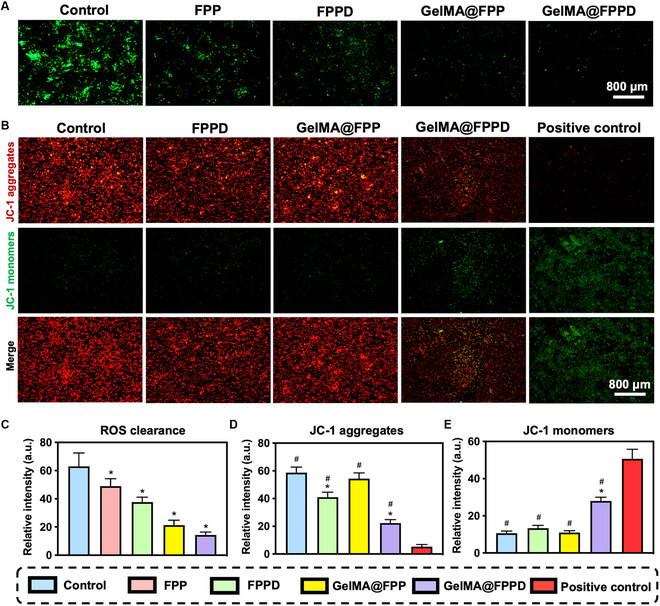
Evaluation of ROS-scavenging and pro-apoptotic capacities of GelMA@FPPD. (A) Representative images of LPS-activated RAW 264.7 macrophages in different groups. Intracellular ROS was stained in green. (B) Representative images demonstrated the mitochondrial membrane potential in different groups. Carbonyl cyanide 3-chlorophenyl hydrazone (CCCP) was utilized to induce mitochondrial dysfunction with a reduction of mitochondrial membrane potential as the positive control. (C) The relative intensity of intracellular ROS revealed by DCFH-DA in different groups. (D) The relative intensity of JC-1 aggregates in each group. (E) The relative intensity of JC-1 monomers in each group (*n* = 3, * and # indicate *P* < 0.05, compared to the control and positive control group, respectively).

However, the synovial fluid system within the knee cavity is dynamic, where small molecular drugs or nanomicelles are prone to be cleared by synovial fluid, diminishing the therapeutic effect [20,48]. Therefore, it is biologically essential to enhance the sustained targeting capacity of the intra-articular drug delivery system. Since M1 macrophage polarization is normally accompanied by excessive ROS production [16], the sustained M1 macrophage-targeted capacity of GelMA@FPPD was evaluated by exploring its ability to scavenge ROS. The intracellular ROS level was assessed by 2',7'-dichlorodihydrofluorescein diacetate (DCFH-DA) fluorescent probe, and the intracellular fluorescence intensity was correlated with the ROS level [49]. Notably, the culture medium was changed at 12 h after nanomicelles or nanomicelle–GelMA microspheres addition, and data were collected at 48 h. Hence, the FPP and FPPD added as pure nanoparticles were cleared due to the medium change, while GelMA@FPP and GelMA@FPPD remained in the system and continued to scavenge ROS by releasing FPP or FPPD nanomicelles, indicating the long-acting regulation capacity of GelMA@FPPD. After lipopolysaccharide (LPS) stimulation, the fluorescence intensity decreased after FPP, FPPD, GelMA@FPP, and GelMA@FPPD treatment compared with the control group (Fig. 5A). Notably, FPP or FPPD loaded on GelMA microspheres exhibited superior therapeutic effects than using FPP or FPPD nanomicelles alone. The fluorescence intensities in FPP and GelMA@FPP groups were 49 ± 5.3 and 21.3 ± 3.5, respectively. Among all groups, the most substantial intracellular ROS level reduction was observed in the GelMA@FPPD group (Fig. 5C). We assumed that these results could be explained by the sustained M1 macrophage-targeted and superior ROS scavenging effects due to the controlled release profile of nanomicelle–GelMA microspheres.

### Evaluation of the capacity of GelMA@FPPD to promote M1 macrophage apoptosis

The inflammatory vicious cycle within the joint could be inhibited by promoting M1 macrophage apoptosis and rebalancing M1/M2 macrophage ratio [50]. In an attempt to verify the capacity of GelMA@FPPD to promote M1 macrophage apoptosis, 5,5′,6,6′-Tetrachloro-1,1′,3,3′-tetraethyl-imidacarbocyanine iodide (JC-1) was utilized to measure the mitochondrial membrane potential (MMP) of M1 macrophages [51]. Macrophages were treated with LPS to promote M1 polarization for 48 h, and another 12 h after nanomicelles or nanomicelle–GelMA microspheres addition, the medium was changed, and data were collected at 48 h. JC-1 would present in cells as aggregates with red fluorescence when MMP was normal. In contrast, JC-1 would present as monomers with green fluorescence when MMP decreased. Therefore, the relative fluorescence intensities of red and green in cells reflected cell pro-apoptotic conditions. Compared with the control group, the intracellular red fluorescence decreased in both FPPD and GelMA@FPPD groups, while green fluorescence increased (Fig. 5B), indicating that both FPPD and GelMA@FPPD promoted M1 macrophage apoptosis, and GelMA@FPPD exhibited a better pro-apoptotic effect on M1 macrophage.

### GelMA@FPPD inhibits M1 macrophage polarization and alleviates “cytokine storm”

**Fig. 6.**
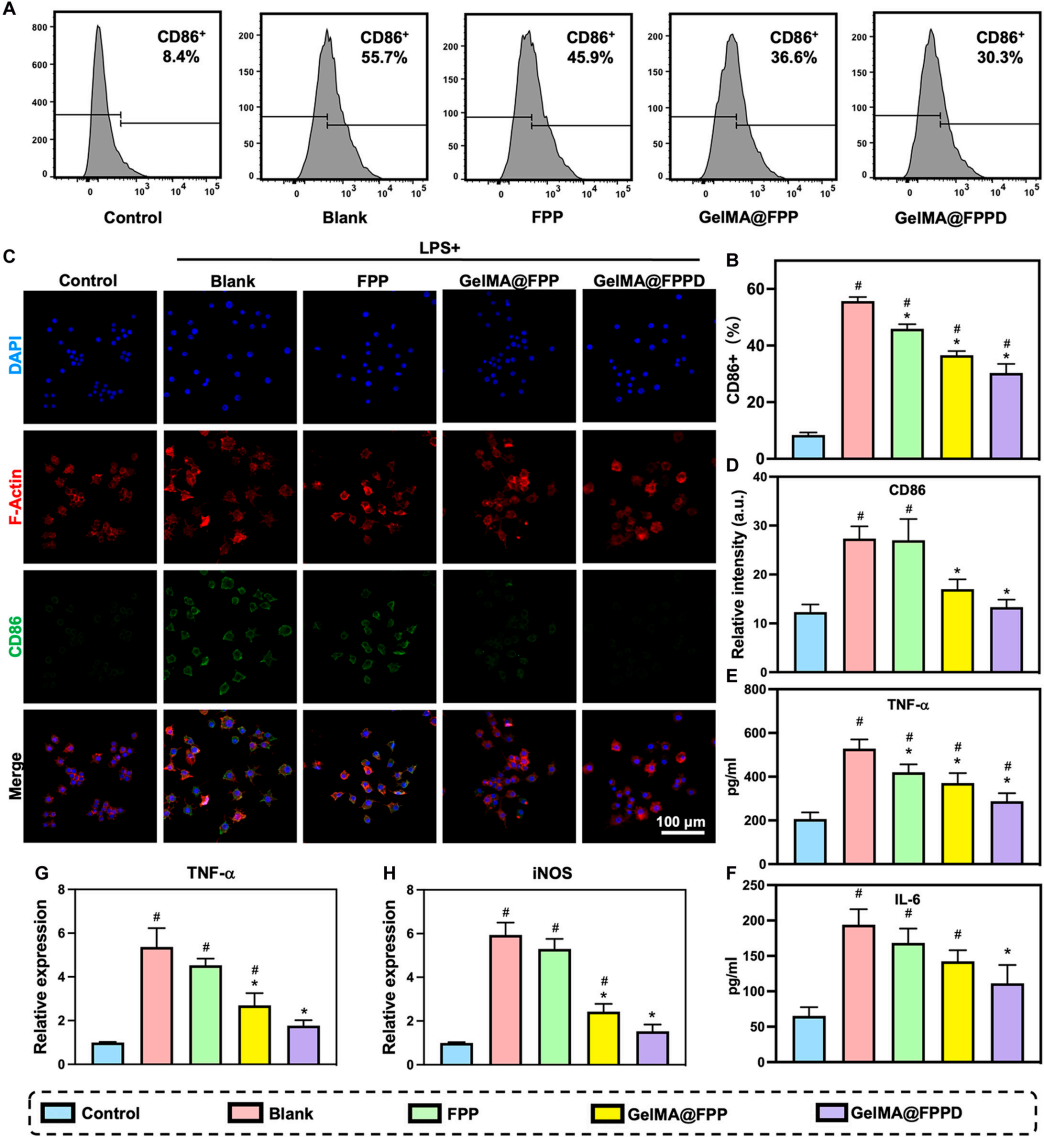
The capacities of GelMA@FPPD to inhibit M1 macrophage polarization and attenuate local “cytokine storm”. (A) Flow cytometric analysis of CD86. (B) The ratios of CD86^+^ RAW 264.7 macrophages in each group. (C) Representative confocal images of LPS-activated RAW 264.7 macrophages in each group. Nucleus: blue; Cytoskeleton: red; CD86: green. (D) Relative fluorescence intensity of CD86 in each group. (E and F) TNF-α and IL-6 measured by ELISA in each group after LPS activation. (G and H) TNF-α and iNOS gene expression levels measured by qRT-PCR in each group after LPS activation (*n* = 3, * and # indicate *P* < 0.05, compared to the control and positive control group, respectively).

M1 macrophage infiltration is anticipated during the acute phase of OA, triggering the “cytokine storm” by producing a large number of inflammatory factors, which accelerates OA progression[28]. Since CD86 was highly expressed on the surface of M1 macrophages [52], the expression level of CD86 on the macrophage cell surface was investigated by flow cytometry and immunofluorescence assays to investigate the capacity of GelMA@FPPD to inhibit M1 macrophage polarization (Fig. 6A). After LPS stimulation, a reduction of CD86 expression was observed in groups treated with nanomicelles or nanomicelle–GelMA microspheres compared to the blank group, and the most substantial decrease was found in the GelMA@FPPD group (Fig. 6B). The immunofluorescence assays showed similar results, with CD86 marked with green fluorescence (Fig. 6C and D). These results suggested that GelMA@FPPD can effectively inhibit M1 macrophage polarization. Theoretically, the reduction of TNF-α, IL-6, and other related inflammatory factors was anticipated by inhibiting M1 macrophage polarization and rebalancing M1/M2 [53]. Therefore, ELISA was utilized to determine TNF-α and IL-6 concentrations and quantitative reverse transcription polymerase chain reaction (qRT-PCR) was further conducted to detect the expression of related inflammatory genes. TNF-α increased from 206 ± 30 pg ml^−1^ to 528 ± 42 pg ml^−1^ after LPS stimulation (*P* < 0.001) and decreased after drug addition with the lowest TNF-α found in the GelMA@FPPD group at 287 ± 37 pg ml^−1^ (Fig. 6E). Meanwhile, similar results were found for IL-6 with the lowest concentration in the GelMA@FPPD group (Fig. 6F).

In addition, qRT-PCR results suggested that the TNF-α gene expression in the blank group after LPS stimulation increased to 5.4 ± 0.9-fold compared to the control group (*P* < 0.001). After treatments with FPPD, GelMA@FPP, and GelMA@FPPD, there was a reduction of the TNF-α gene expression with the most substantial plunge in the GelMA@FPPD group at 1.8 ± 0.3-fold compared to the control group (*P* < 0.001) (Fig. 6G). iNOS gene expression levels demonstrated a similar trend (Fig. 6H). Compared to the control group (1.0 ± 0.03), iNOS gene expression was 5.9 ± 0.6-fold in the blank group (*P* < 0.001) and 1.5 ± 0.3-fold in the GelMA@FPPD group (*P* < 0.001). These results indicated that by sustained macrophage targeting, GelMA@FPPD could inhibit M1 polarization, downregulate inflammatory factors, and alleviate local “cytokine storm”.

### In situ sustained treatment of nanomicelle–GelMA microspheres

**Fig. 7.**
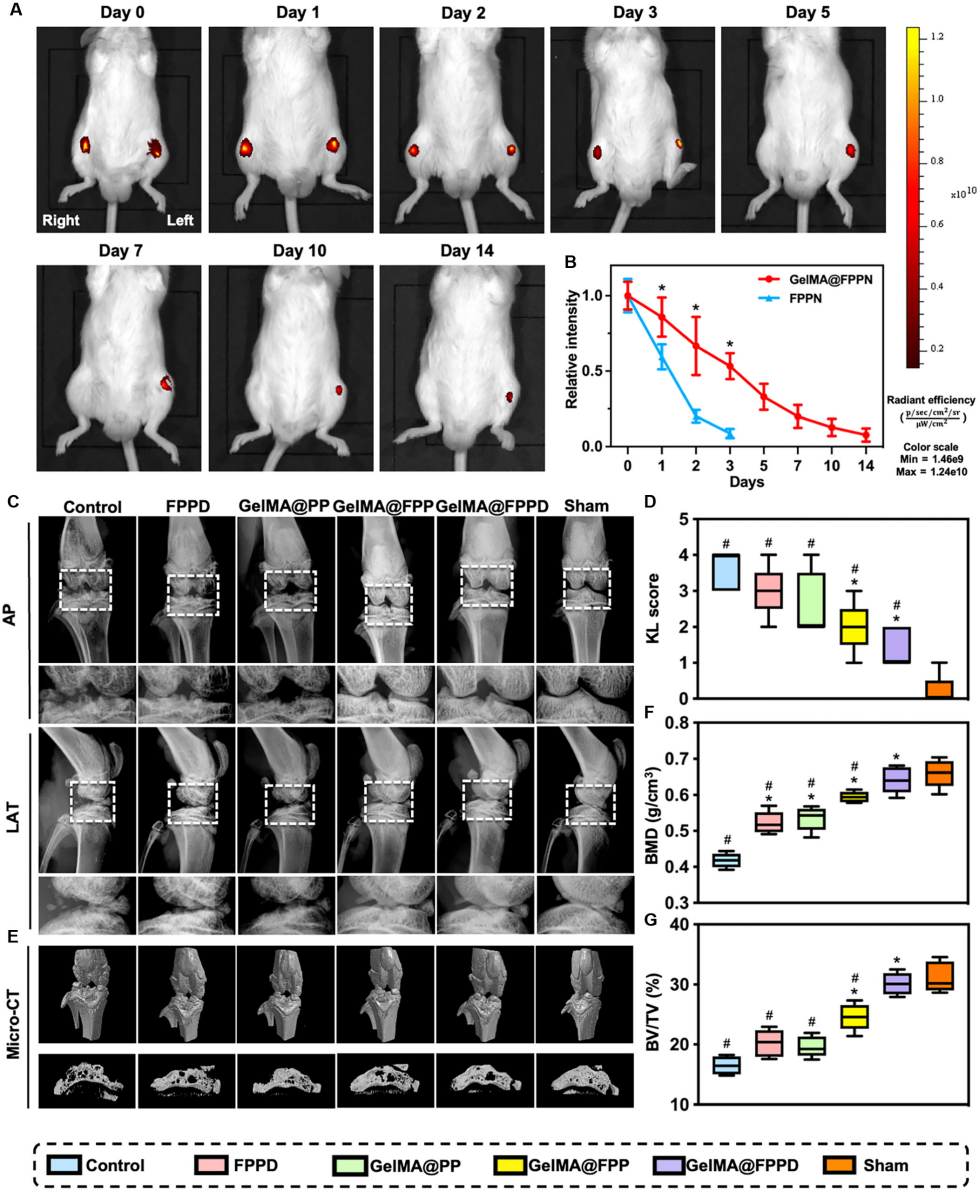
IVIS and radiographic evaluations. (A) Representative IVIS images of Nile Red-labeled nanomicelles remained in knee joints at designated time points. GelMA@FPPN were injected into the left knee joint, while FPPN nanomicelles were injected into the right knee. Images were taken with the ICR mice placed in supine position. (B) Relative fluorescence intensity of the left and right knee joints at different time points (*n* = 3). (C) Representative radiographic images of the knee joints in different groups. AP: anterior–posterior; LAT: lateral. (D) Kellgren–Lawrence OA scores were measured in each group. (E) Representative micro-CT images in each group. (F and G) BMD and BV/TV of the proximal tibial subchondral bone were analyzed according to micro-CT results in each group (*n* = 5) (* and # indicate *P* < 0.05, compared to the control and positive control group, respectively).

Prolonging the retention time of nanomicelles within the joint cavity is of paramount importance to enable in situ sustained treatment by maintaining therapeutic drug concentration, thus facilitating OA treatment. Therefore, the in situ sustained therapeutic efficacy of GelMA@FPPD was validated by the in vivo imaging system (IVIS). Nile Red-labeled nanomicelles (FPP@Nile Red, FPPN) and nanomicelle–GelMA microspheres (GelMA@FPP@Nile Red, GelMA@FPPN) were fabricated. GelMA@FPPN was injected into the left knee of ICR mice, while FPPN nanomicelles were injected into the right knee. The relative fluorescence intensity of the right knee declined rapidly to 8.7 ± 3% on day 3 and almost vanished on day 5 (Fig. 7A and B). The fluorescence intensity of the left knee existed for a longer time and diminished more slowly than the right knee, with the relative fluorescence intensity of 53.3 ± 8.6% on day 3, 19.9 ± 7.7% on day 7, and 7.7 ± 4.3% on day 14. The results suggested that FPPN, which entered the joint cavity as nanomicelles, was rapidly cleared within 3 days due to synovial fluid exchange. In contrast, the fluorescence intensity of GelMA@FPPN was maintained for 14 days, indicating that GelMA microspheres could provide excellent physical protection for the encapsulated nanomicelles and maintain their controlled release, which was assumed to be essential for the in situ sustained treatment of OA.

### Evaluation of cartilage protection property of GelMA@FPPD in rat OA

#### Radiographic evaluation

OA microenvironment was detrimental to chondrocytes’ physiological function, inhibiting their synthesis of extracellular matrix (ECM) and disrupting the cartilage structure [54–56]. In order to validate the cartilage protection property of GelMA@FPPD in vivo, the rat OA model was established by intra-articular injection of 5 mg kg^−1^ of sodium iodoacetate (MIA) into the knee joint of Sprague–Dawley rats [57]. After 2 weeks of OA induction, the OA rats were further treated with nanomicelles or nanomicelle–GelMA microspheres intra-articularly. OA severity was evaluated using x-rays at 8 weeks after the establishment of OA. X-ray results showed that rats in the control group demonstrated the most severe knee OA with massive osteophyte formation, subchondral bone destruction, marked joint space narrowing, and osteoporosis of the epiphysis (Fig. 7C and D). In contrast, GelMA@FPP and GelMA@FPPD exhibited substantial therapeutic effects on OA, with mild narrowing of the joint space, few osteophyte formation, and relatively dense subchondral bone. Besides, the Kellgren–Lawrence score confirmed that GelMA@FPPD was superior to other groups in alleviating OA.

In order to further investigate the subchondral bone of knee joints, micro-CT scans were conducted. The lowest bone mineral density (BMD) of the proximal tibia was observed in the control group (0.42 ± 0.02 g/cm^3^), while BMDs in other treatment groups were significantly higher compared to the control group, with the highest BMD in the GelMA@FPPD group (0.64 ± 0.04 g/cm^3^, *P* < 0.001) (Fig. 7E and F). The bone volume fraction (BV/TV), which reflected bone volume change, was also investigated. The BV/TV in the GelMA@FPPD group (30.1 ± 1.8%) was significantly higher than that in the control group (16.5 ± 1.5%) (*P* < 0.001) (Fig. 7G). These results suggested that compared to other groups, GelMA@FPPD showed the most prominent advantage in protecting cartilage and subchondral bone and alleviating OA progression.

#### Gross appearance evaluation

**Fig. 8.**
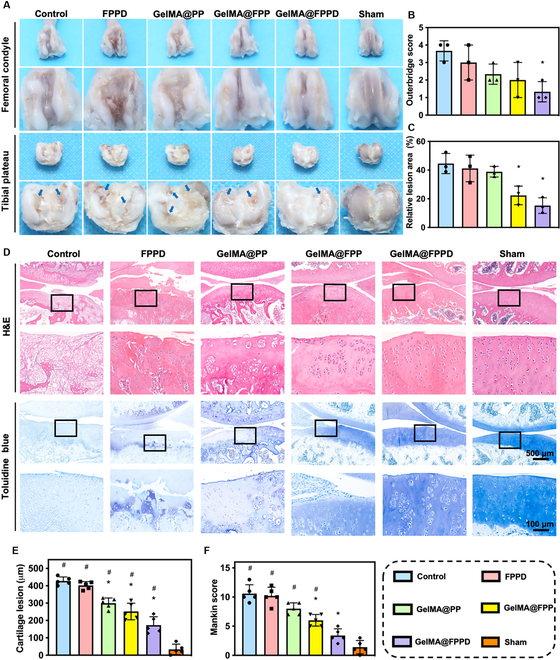
(A) Gross appearance evaluation of the knee specimens. Representative images of the articular surfaces of the femoral condyle and tibial plateau in each group. Blue arrows indicate cartilage defects. (B and C) The Outerbridge score and relative lesion area were analyzed based on the direct investigation of gross appearance in each group (*n* = 3). (D) Representative H&E and toluidine blue staining images in each group. (E and F) Cartilage lesion depth and total Mankin score were analyzed according to the staining images in each group (*n* = 5) (* and # indicate *P* < 0.05, compared to the control and positive control group, respectively).

In an attempt to further investigate the protective effect of GelMA@FPPD on knee cartilage in the rat OA model, the gross appearance of the knee joints was evaluated by direct visualization. The Outerbridge scores were 3.7 ± 0.6 and 3.0 ± 1.0 points in control and FPPD groups, respectively (*P* = 0.73), with marked cartilage destruction and fibrosis of tibial plateau articular surface, exposure and fissure formation of subchondral bone, and extensive osteophyte formation (Fig. 8A and B). The Outerbridge scores were 2.3 ± 0.6 and 2.0 ± 1.0 points in the GelMA@PP and GelMA@FPP groups, with improvements in cartilage destruction compared to the control group. Notably, the Outerbridge score in the GelMA@FPPD group (1.3 ± 0.6 points) was significantly improved compared to the control group (3.7 ± 0.6 points) (*P* = 0.027 < 0.05), with considerable amelioration of articular cartilage condition. In addition, the relative area of damaged tibial plateau cartilage was also measured in this study. The greatest damaged area was found in the control group (44.5 ± 7.0%), and improvements in other groups were observed with the therapeutic effects ranked as GelMA@FPPD > GelMA@FPP > GelMA@PP > FPPD (Fig. 8C). These results indicated that the GelMA@FPPD presented the most considerable cartilage protection property in the rat OA model due to the capacity to maintain sustained release of FPPD nanomicelles from GelMA microspheres within the joint cavity, achieving the in situ long-acting targeting and modulation of M1 macrophages. The IVIS results confirmed that the fluorescence intensity diminished at 3 days after intra-articular injection of nanomicelles alone, whereas nanomicelle–GelMA microspheres could considerably maintain fluorescence intensity for 14 days (Fig. 7). Since drugs were delivered intra-articularly at 14-day intervals after the establishment of OA, rapid clearance of injected FPPD nanomicelles was expected, hindering the maintenance of sufficient therapeutic drug concentration. In contrast, owing to the protective effect of GelMA microspheres and their capacity to maintain controlled release of encapsulated FPPD nanomicelles for 14 days, we assumed that GelMA@FPPD could manage to alleviate local “cytokine storm”, regulate inflammation, and delay OA progression by in situ sustained M1 macrophage targeting and M1/M2 rebalance.

#### Histological evaluation

Hematoxylin–eosin (H&E), toluidine blue, Safranin O/fast green, and immunofluorescence staining of type II collagen were carried out to further evaluate the therapeutic efficacy of GelMA@FPPD in the rat OA model. H&E and toluidine blue staining (Fig. 8D to F) suggested that considerable cartilage destruction was identified in control and FPPD groups, with severe cartilage damage involving almost the entire cartilage depth to the subchondral bone, disorganization of embedded chondrocytes and surrounding ECM, hypocellularity, and reduction in staining. The depth of damaged cartilage was 428 ± 23 μm in the control group and 402 ± 22 μm in the FPPD group (*P* = 0.866), with total Mankin scores of 10.6 ± 1.5 and 10.2 ± 1.5 points, respectively (*P* = 0.996). The limited cartilage protective effect of FPPD was related to the rapid clearance of FPPD nanomicelles within the knee joint. In contrast, amelioration of cartilage destruction in GelMA@PP and GelMA@FPP groups was observed, with reduced extent and depth of cartilage involvement and increased chondrocytes in clusters and staining improvement. Notably, with slight cartilage involvement localized in the superficial layer, slight disorganization of chondrocytes, and nearly normal staining, the GelMA@FPPD group displayed the most substantial therapeutic efficacy due to its in situ long-acting macrophage-targeted capacity.

**Fig. 9.**
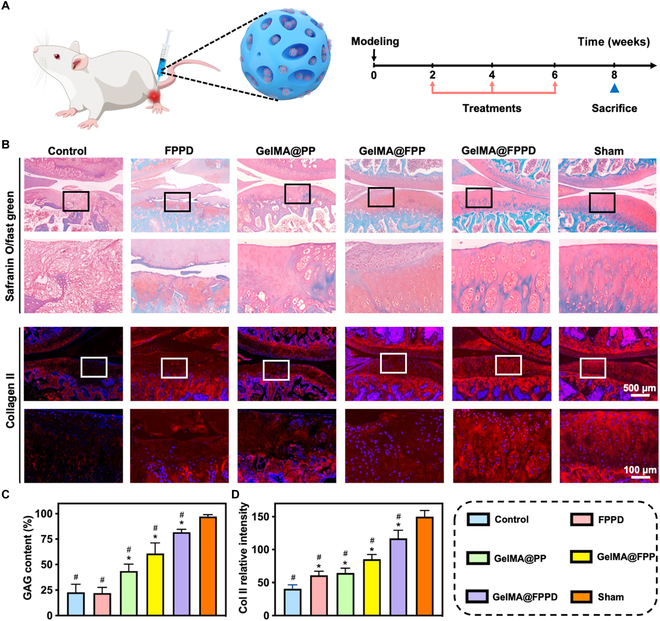
(A) Schematic diagram demonstrated that GelMA@FPPD were delivered intra-articularly for OA treatment and the timeline for the animal model. (B) Representative images of Safranin O/fast green and type II collagen immunofluorescence staining in each group. (C and D) Relative GAG content and type II collagen expression were analyzed in each group. Nucleus: blue; Type II collagen: red (*n* = 5, * and # indicate *P* < 0.05, compared to the control and positive control group, respectively).

Glycosaminoglycan (GAG) and type II collagen are 2 major components of ECM. A higher content of type II collagen and GAG represents better cartilage condition. The relative GAG content was 22.8 ± 8.0% in the control group and 24.0 ± 4.6% in the FPPD group (Fig. 9A to C). Compared to the control group, the relative GAG contents were significantly higher at 43.8 ± 6.7% in the GelMA@PP group (*P* < 0.001), 60.8 ± 10.4% in the GelMA@FPP group (*P* < 0.001), and 81.8 ± 2.9% in the GelMA@FPPD group (*P* < 0.001). Meanwhile, similar results were found regarding the contents of type II collagen in different groups, with the highest content identified in the GelMA@FPPD group. These histological findings validated the assumption that encapsulation of FPPD nanomicelles in GelMA microspheres could help achieve in situ sustained targeting when delivered intra-articularly, enabling its superior therapeutic efficacy in OA.

### Biosafety of GelMA@FPPD

The biosafety of the drug delivery system was a prerequisite for any medical treatment. The toxic effects on vital systemic organs, including the heart, liver, spleen, lung, and kidney, were evaluated in animal experiments. H&E staining of these organs suggested no differences between sham and other groups, indicating that the drug delivery system in the present study was biologically safe and had no toxic effects on vital organs (Fig. S3).

## Conclusions

In this study, in situ sustained macrophage-targeted nanomicelle–hydrogel microspheres (GelMA@FPPD) were successfully constructed via microfluidic technology and nanotechnology. The porous structure of GelMA microspheres enabled the controlled release of encapsulated FPPD nanomicelles while preventing the rapid clearance triggered by the joint fluid exchange. Moreover, the specific binding interaction between FA on the surface of FPPD nanomicelles and FA receptors expressed on M1 macrophage cell surface enhanced the subsequent uptake of DEX-loaded FPPD nanomicelles, alleviating OA by in situ sustained macrophage targeting and regulation. In vitro and in vivo experiments validated that this novel drug delivery system could promote M1 macrophage apoptosis, inhibit M1 polarization, and downregulate inflammatory factors secreted by M1 macrophages, thereby inhibiting the vicious cycle of “cytokine storm”, thus effectively protecting inflamed articular cartilage and alleviating OA progression. This novel macrophage-targeted drug delivery system could endow great potential in the treatment of macrophage-mediated diseases.

## Materials and Methods

All experimental details are reported in the Supplementary Materials. All animal experiments were approved by the Animal Research Committee of Ruijin Hospital, School of Medicine, Shanghai Jiao Tong University, approval number: SYXK 018-0027.

## Data Availability

The data used to support the findings of this study are present in the paper and supporting materials. Additional data related to this paper may be requested from the authors.

## Supplementary Materials

Supplementary Materials Experimental Section Figs. S1 to S3 Table S1
