# Supplementary material for: In Situ Sustained Macrophage-Targeted Nanomicelle–Hydrogel Microspheres for Inhibiting Osteoarthritis
Source: Research (Wash D C). 2023 May 2;6:0131. doi: 10.34133/research.0131 (PMC10202383; doi:10.34133/research.0131)
Supplement: Supplementary Materials — Experimental Section Figs. S1 to S3 Table S1 [file research.0131.f1.docx]

Supporting Information

*In situ* sustained macrophage-targeted nanomicelle-hydrogel microspheres for inhibiting osteoarthritis

XiaoXiao Li^†^, Xingchen Li^†^, Jielai Yang^†^, Yawei Du, Liang Chen, Gang Zhao, Tingjun Ye, Yuan Zhu, Xiangyang Xu, Linfu Deng and Wenguo Cui*

Experimental Section

***Materials:*** Propylene sulfide (PS), 1,8-Diazabicyclo[5.4.0]undec-7-ene (DBU), 1-Butane thiol, Iodoacetic acid, Tetrahydrofuran (THF), N,N-Dimethylformamide (DMF), Dimethyl sulfoxide (DMSO), 1-(3-Dimethylaminopropyl)-3-ethylcarbodiimide hydrochloride (EDC), 4-Dimethylaminopyridine (DMAP), Folic acid (FA), Dexamethasone (DEX), Lithium phenyl-2,4,6-trimethylbenzoylphosphinate (LAP), methacrylic anhydride (MA) were obtained from Aladdin. Gelatin was purchased from Macklin. NH_2_-PEG-NH_2_ was purchased from Xi’an Qiyue Biological Technology Co., Ltd.

***Polymer synthesis and characterization:*** I) Synthesis of PEG-PPS block polymer (PP polymer) PEG-PPS: 1-butane thiol (2.5 mmol, 0.27 mL) in THF (30 mL) was added dropwise into the DBU solution (7.5 mmol, 1.1 mL) (in 30 mL of dry THF) under a nitrogen atmosphere at 0 °C. Later, freshly distilled and degassed PS (200 mmol, 15.7 mL) was added and allowed to react for 2 h. The reaction was quenched by adding Iodoacetic acid, and the resultant PPS-COOH polymer was filtered and washed with cold methanol before vacuum-drying. Certain amounts of NH_2_-PEG-NH_2_, EDC, and DMAP were dissolved in 20 mL of DMF. The prepared PPS-COOH in DMF was then added to the reaction mixture, stirring for 48 h. The resultant PEG-PPS (PP) was dialyzed (MWCO=2000) before lyophilization. The chemical structure of purified PP block polymer was analyzed by ^1^H NMR (400 mHZ, Avance Ⅲ, Germany). II) Synthesis of FA-PEG-PPS block polymer (FPP polymer): The prepared PP block polymer was dissolved in DMSO, followed by EDC and DMAP. FA was added, and the reaction was stirred at 30 °C for 48 h. The resulting products were dialyzed (MWCO=3500) and lyophilized to obtain the FA-PEG-PPS (FPP) product. The chemical structure of purified FPP block polymer was analyzed by ^1^H NMR.

***Preparation and characterization of PP or FPP nanomicelles:*** To prepare nanomicelle solutions, 10 mg of purified PP and FPP block polymer was separately dissolved in THF. The block polymer solution (in THF) was then slowly added dropwise into 1 mL of PBS, and the mixture was stirred at 40 °C for 24 h to remove THF. The obtained nanomicelles were denoted as PP nanomicelles and FPP nanomicelles, respectively, depending on the usage of the polymer. Dynamic light scattering (DLS, ZetaSizer Nano ZS, UK) was conducted to measure the hydrodynamic diameter with different media (PBS, water). Transmission electron microscopy (TEM, Talos F200X G2, US) was used to observe the micelle morphology.

***DEX-loaded nanomicelles:*** 10 mg of PP or FPP block polymer was dissolved in THF, followed by different amounts of DEX. The mixture was dropwise into 1 mL of PBS and stirred at 40 °C for 24 h to remove THF. Finally, the mixture was centrifuged (2000 r min^-1^, 5 min) to remove the unloaded DEX. The DEX-loaded nanomicelles were denoted as PPD and FPPD nanomicelles depending on the usage of PEG-PPS or FA-PEG-PPS polymer. The unloaded DEX was redispersed into 75% ethanol solution, in which the absorbance was detected by UV-vis spectrophotometer (Eppendorf, Germany) at a wavelength of 240 nm. The drug loading capacity (LC%) and loading efficiency (LE%) were calculated by the modified equation:

$$LC (\%)=\frac{W_{t}}{W_{0}}100\%$$

$$LE (\%)=\frac{W_{t}}{W_{nanomicelles}+W_{t}}100\%$$

Where W_t_ is the loaded weight of DEX in nanomicelles calculated by UV-vis data, W_0_ is the initial feeding weight of DEX, and W_nanomicelles_ is the weight of FA/PEG-PPS.

***Microsphere preparation and characterization:*** I) Synthesis of GelMA monomer: GelMA monomer was synthesized based on slight modifications from previous studies.^1^ Briefly, 50 g of Gelatin was dissolved in 500 mL of PBS. And 16 mL of MA was slowly injected into the mixture by syringe pumps (Lead Fluid, China). The reaction lasted 2 h, and the resulting GelMA solution was dialyzed against deionized water before lyophilization. II) Preparation of nanomicelles loaded GelMA microspheres: PPD or FPPD nanomicelles loaded GelMA microspheres were prepared using the microfluidic technology described in previous studies.^2, 3^ The water phase was composed of 0.5% LAP and 5% GelMA-nanomicelles solution (0.5 g of GelMA dissolved in 10 mL of 10 mg mL^-1^ PPD or FPPD nanomicelles solution). The oil phase was composed of paraffin oil and 5% Span 80. The oil phase could continuously cut the water phase so that GelMA-nanomicelles formed droplets which were transferred to the frozen Petri dish (-40 °C), followed by photo-cross-linked under ultraviolet radiation (365 nm, 30 min). The crosslinked microspheres were washed with 75% ethanol and acetone before lyophilization. And the resulting microspheres were denoted as GelMA@PPD and GelMA@FPPD, depending on the type of nanomicelles.

***ROS-mediated release of DEX:*** 1 mL of FPPD nanomicelles or GelMA@FPPD microspheres was transferred into a dialysis bag (MWCO=3500) and submerged into 9 mL of PBS or 1 mM H_2_O_2_ solution (37 ^o^C, pH 7.4). At each designated time points, 1 mL of dialysate was taken as a sample, and 1 mL of PBS or 1 mM H_2_O_2_ solution was refilled. The concentration of DEX that was released into the test medium was obtained by quantifying the absorption of DEX using a UV-vis spectrophotometer.

***Degradation property of GelMA microspheres:*** To investigate the degradation profile, the fresh GelMA@FPPD microspheres were placed in a degradation solution (PBS (pH=7.4) containing 0.1 U mL^-1^ of collagenase II). And the samples were being agitated in a shaker at 37 °C, 80 rpm. The solution was refreshed every three days, and the microspheres were observed at different time points to assess the morphological changes. The degradation profile was quantified by the following steps: Briefly, 10 mg of lyophilized GelMA@FPPD microspheres were placed in EP tubes and immersed in the above degradation solution. The samples were collected at predetermined time points and further lyophilized to obtain the total mass (m_1_). The residual percentage (RP) was calculated as the following equation:

*RP (%) =*$\frac{\text{m}_{\text{1}}\text{-}\text{m}_{\text{0}}}{\text{10}\text{ }}\text{×100\%}$

(m_1_ refers to the total mass of each residual lyophilized microspheres and EP tube. m_0_ refers to the mass of each empty EP tube.)

***Cell culture:*** The macrophages used for cell experiments in this study were RAW 264.7 (Procell CL-0190), which were kindly provided by Procell Life Science & Technology Co., Ltd. Unless mentioned elsewhere, all of the other reagents used for cell experiments were purchased from Gibco (USA). Dulbecco’s Modified Eagle Medium (DMEM)/high glucose with 10% fetal bovine serum, 100 U mL^-1^ penicillin, and 100 g mL^-1^ streptomycin at 37 ℃ with 5% CO_2_ were used for cell culture. The cell growth was observed under bright field microscope. When the cell growth density reached 80%-90% confluence, the cells were passaged at 1:4 or 1:5. The RAW 264.7 cells grew in a semi-adherent state in the culture dish. When passaged, part of the medium was discarded, and the cells could be dislodged gently and then inoculated into the culture dish containing fresh medium. The RAW264.7 cell line proliferates rapidly and needs to be passaged every 2-3 days.

***Cytocompatibility of GelMA@FPPD:*** I) Live/dead staining: RAW 264.7 cells were inoculated into 12-well plates at 1×10^4^/well and incubated in the cell culture incubator for 4 h, and then GelMA microspheres, PP or FPP nanomicelles, and GelMA@FPPD microspheres were added to the plates in different groups. When GelMA microspheres were added, Transwell co-culture system (0.4 μm, Corning, USA) was used, and microspheres were inoculated in the upper compartment of the Transwell, isolating from the macrophages in the lower compartment while allowing drugs to diffuse freely through the micropores on the membrane. On day 1, 2, and 3, the medium was discarded, and cells were washed with PBS three times. Live/dead staining reagent (Beyotime, China) was added for 20 min, and live cells (green) and dead cells (red) were then observed under a fluorescence microscope (Zeiss, Germany). The live cells were counted using ImageJ software subsequently.

II) Cell count kit-8 assay (CCK8): RAW 264.7 cells were inoculated at 2×10^3^/well into the lower chamber of Transwell 96-well plates (0.4 μm, Corning, USA), and different drugs were added to the upper chamber in different groups. On days 1, 2, and 3, CCK8 reagent (Beyotime, China) was added and incubated for 2 h. The optical density (OD) values were then obtained at 450 nm using microplate reader (BioTek, USA).

Ⅲ) Hemolytic activity: Fresh blood was taken from SD rats and the flow cytometry tubes were washed three times with normal saline. The positive control group was added with 2 mL sterilized deionized water, while the other groups were added with 2 mL normal saline. Biomaterials (10 mg ml^-1^ of FPPD, 5 wt% GelMA, GelMA@FPPD (containing 10 mg ml^-1^  FPPD) were added based on different groups and incubated at 37 °C for 1 h. Then, 120 µL fresh blood was added to each tube and incubated at 37 °C for another 1 h. 1 mL of the above mixtures were transferred to a 2 mL centrifuge tube, centrifuged at 2500 rpm for 5 min, and then photographed. 200 µL supernatants were taken and added in a 96-well plate. OD values were measured at 545 nm wavelength with microplate reader (BioTek, USA).

The hemolytic ratio (%) = $\frac{{OD}_{sample}-{OD}_{negative}}{{OD}_{positive}-{OD}_{negative}}*100\%$

***Targeting capability of FPP nanomicelles:*** FPP@Nile Red (FPPN) and PP@Nile Red (PPN) nanomicelles were prepared as described above. RAW 264.7 cells were inoculated into 12-well plates at 5×10^4^/well, and 5 g mL^-1^ lipopolysaccharide (LPS, Solarbio, China) was added to each well the next day to activate macrophages for another 48 h. Then FPPN and PPN nanomicelles were added separately, and then the medium was discarded and washed three times with PBS at 0, 15, 30, 60 and 120 min. The cells were then fixed with 4% paraformaldehyde for 20 min and finally stained with the cell nuclear dye DAPI (Beyotime, China) for 10 min. FPPN and PPN in the cells were observed under confocal microscopy (Zeiss, Germany) with Ex/Em=530/635 nm. Images were taken and analyzed using ImageJ software.

***ROS scavenging capacity:*** RAW 264.7 cells were inoculated into 12-well plates at 5×10^4^/well and were added with 5 μg mL^-1^ LPS, along with FPP, FPPD, GelMA@FPP, and GelMA@FPPD in different groups. Similarly, the Transwell co-culture system (0.4 μm, Corning, USA) was utilized to inoculate microspheres into the upper compartment. To simulate *in vivo* clearance of nanomicelles, the medium was changed after 12 h of co-culture. After co-culture for 48 h, the culture medium was discarded, and the cells were washed 3 times with PBS and co-cultured for 20 min with serum-free DMEM diluted 2',7'-dichlorodihydrofluorescein diacetate (DCFH-DA, Beyotime, China). The intracellular green fluorescence intensity was observed at Ex/Em=488/525 nm and then photographed and analyzed using ImageJ software.

***Mitochondrial membrane potential assessment:*** RAW 264.7 cells were inoculated into 12-well plates at 2×10^4^/well and were stimulated with 5 μg mL^-1^ LPS for 48 h the next day, followed by the addition of FPPD, GelMA@FPP, and GelMA@FPPD in different groups. Similarly, the Transwell co-culture system (0.4 μm, Corning, USA) was used for GelMA microspheres. The medium was changed after 12 h of co-culture and discarded after 36 h. The cells were rinsed with PBS, serum-free DMEM was added, and then JC-1 dye (Beyotime, China) was added and observed under a fluorescence microscope after 20 min of co-incubation. The relative intensity of fluorescence was analyzed using ImageJ software.

**GelMA@FPPD inhibits M1 macrophage polarization:** I) Flow cytometry: RAW 264.7 cells were inoculated into 6-well plates at 1×10^5^/well and were stimulated with 5 μg mL^-1^ LPS for 48 h the next day, followed by the addition of FPPD, GelMA@FPP, and GelMA@FPPD in different groups. Similarly, the Transwell co-culture system (0.4 μm, Corning, USA) was used for GelMA microspheres. The cells were incubated in the incubator for 12 h. After changing the medium, the co-culture was continued for another 48 h. The adherent cells were gently blown and transferred to 1.5 mL Eppendorf (EP) tubes, and the supernatant was removed by centrifugation (1000 rpm, 5 min). The cells were washed with 1 mL PBS, and CD86 monoclonal antibody (Biolegend, USA) was added and incubated for 40 min at 4 °C. 1 mL flow staining buffer was added, and the cells were resuspended. 500 μL of flow staining buffer was added after centrifugation, and then the cell solution was transferred for flow cytometry analysis (BD FACSAria III, USA). II) Immunofluorescence staining: RAW 264.7 cells were inoculated into 24-well plates at 2×10^4^/well and were added with LPS and different drugs as described in I) flow cytometry. After 48 h of co-culture, the medium was discarded, rinsed three times with PBS, and fixed with 4% paraformaldehyde for 20 min. 5% skimmed milk was used to block the cells for 2 h in a constant temperature shaker at 4 °C. After rinsing the cells three times with PBS, Mouse CD86 antibody (Affinity, USA) diluted at 1:200 was added and incubated overnight at 4 ℃ in a constant temperature shaker. The next day, the cells were rinsed 3 times again with PBS and added with the secondary antibody (Abcam, USA) diluted at 1:500. The secondary antibodies were protected from light during addition and incubated for one hour at 4 ºC in a shaker followed by three rinses of PBS. Finally, the cytoskeleton and nuclei were stained with phalloidin (Kingmorn, China) and DAPI (Beyotime, China). Confocal microscopy was utilized for observation and the immunofluorescence images were further analyzed by ImageJ software. III) Elisa: As described in I) flow cytometry, RAW 264.7 cells were inoculated in a 6-well plate, LPS and different drugs were added sequentially, and the upper layer of the culture medium was removed after 48 h co-culture. The concentrations of TNF-α and IL-6 were detected using Elisa kits (MLBIO, China), and the OD values were measured at 450 nm using microplate reader. IV) qRT-PCR: As described in I) flow cytometry, RAW 264.7 cells were inoculated in a 6-well plate, LPS and different drugs were added sequentially, and the medium was removed after 48 h co-culture. After cell lysis by adding RNA extraction solution (Takara, Japan), RNA was extracted and measured using trichloromethane and isopropanol, then reversely transcribed to cDNA using a reverse transcription kit (Takara, Japan). Then cDNA was amplified using the qRT-PCR kit (Takara, Japan) (LightCycle@96, Switzerland). The expressions of the following three genes, β-actin, TNF-α and iNOS, were calculated *via* ΔΔCT method. β-actin was utilized as the reference gene. The primer sequences of the above three genes were listed in **Table S1**.

***Controlled release of FPP nanomicelles from GelMA microspheres:*** IVIS (Xenogen, USA) was utilized to evaluate the capacity of GelMA microspheres to maintain controlled release of encapsulated nanomicelles. Firstly, FPPN nanomicelle and FPPN nanomicelle loaded GelMA microspheres (GelMA@FPPN), were prepared as described previously. GelMA@FPPN and FPPN nanomicelle solution that contained equal amounts of FPPN were injected into the knee joints of ICR mice, with GelMA@FPPN injected into the left knee joint and FPPN nanomicelles injected into the right knee joint (injection volume=100 μL). The fluorescence intensity of the knee joint was detected using IVIS on day 0, 1, 2, 3, 5, 7, 10, and 14, respectively, with Ex/Em=530/635 nm. The relative fluorescence intensities at different time points were recorded with the intensity on day 0 as reference.

***The capacity of GelMA@FPPD to alleviate OA progression:*** I) Establishment of OA model in SD rats: The 8-week-old male *Sprague Dawley* (SD) rats (mean weight 200-220 g) were used. The rats were anesthetized by intraperitoneal injection of 3% sodium pentobarbital at 40 mg kg^-1^. OA was induced by intra-articular injection of 5 mg kg^-1^ sodium iodoacetate (MIA) into the knee joint immediately after anesthesia.^4^ The right knee was selected as the experimental side, and the sham group was treated with PBS (pH=7.4). The animals were divided into 6 groups: sham, control, FPPD, GelMA@PP, GelMA@FPP, and GelMA@FPPD. Animals were treated regularly by intra-articular injection at week 2, 4, and 6 after model establishment. The injection volume was 200 μL. The sham group did not receive any intra-articular injection and the control group was injected with PBS (pH=7.4). II): X-ray and micro-CT evaluation of rat knees: The knee joints were harvested at week 8 after surgery. Briefly, rats were euthanized by cervical dislocation after anesthesia. The muscle around the knee joint was removed, preserving the knee capsule's integrity. The anterior-posterior and lateral views of X-rays (Faxitron X-ray, USA) were obtained in all knee joints, followed by fixation in a 4% paraformaldehyde solution. The OA severity of the knee joint in different groups was analyzed by the Kellgren-Lawrence (KL) OA score. The KL score ranged from 0-4, representing 5 different levels of OA severity, with 0 being no OA and 1-4 being mild, moderate, severe, and very severe OA, respectively. In addition, micro-CT (Skyscan1076, Bruker, Belgium) of the knee was performed to assess the subchondral bone condition. The bone mineralization density (BMD) and the subchondral bone volume fraction (BV/TV) of the proximal tibia were further analyzed. III): Gross appearance evaluation of rat knees: Anatomic assessments of the knee joint in SD rats were performed in this study to determine the therapeutic efficacy of GelMA@FPPD on OA. After carefully removing the muscles, ligaments, and meniscal tissues around the knee joint, the femoral condyle was separated from the tibial plateau, and the gross appearance of articular cartilage was evaluated under direct visualization. The Outerbridge score was utilized to quantify articular cartilage destruction,^5^ with 0 indicating normal, smooth, and white in color of the articular surface, 1 indicating good cartilage integrity but edema and softening of the cartilage surface with localized color changes, 2 indicating incomplete cartilage, with fissures, fibrosis and exfoliation of the superficial cartilage, 3 indicating cartilage fissures involving the subchondral bone, with partial loss and exfoliation of the cartilage, and 4 indicating exposed subchondral bone, with significant cartilage erosion, fibrosis, and exfoliation. In addition, the specimens were photographed and analyzed for the relative area of cartilage damage, which was measured as the percentage of damaged cartilage area to the entire tibial plateau area using ImageJ software. IV) Pathological and immunofluorescence staining: The knee joint, heart, liver, spleen, lung, and kidney were harvested in different groups and then fixed in 4% paraformaldehyde solution. The knee joint specimens were decalcified for 8 weeks, paraffin-embedded, and sectioned with a layer thickness of 5 μm. The tissue sections were stained with H&E, Safranin O, and toluidine blue, and the degeneration of articular cartilage was observed and evaluated under bright-field microscopy. The depth of damaged cartilage was then measured from the tissue sections, along with the Mankin score^6^ and relative GAG content. Visceral tissues were directly stained by H&E after paraffin embedding and sectioning. For type II collagen immunofluorescence staining, after the sections were dewaxed, the antigen was repaired, then the autofluorescence was quenched and closed with BSA for 30 min. The primary and secondary antibodies (Servicebio, China) of type II collagen were added sequentially, and the nuclei were further stained with DAPI. The nuclei appeared blue and type II collagen appeared red. The expression of type II collagen in cartilage tissue was evaluated under a fluorescence microscope, and analyzed using ImageJ software.

***Statistical analysis:*** All experimental data were presented as mean ± standard deviation. Graphpad Prism 9.3.1 was utilized for data collection, statistical analysis and graph drawing, student’s t-test and Tukey’s multiple comparisons test were conducted to investigate the differences among groups. p<0.05 was considered as statistically significant. The relative fluorescence intensities were measured by ImageJ software 2.9.0.


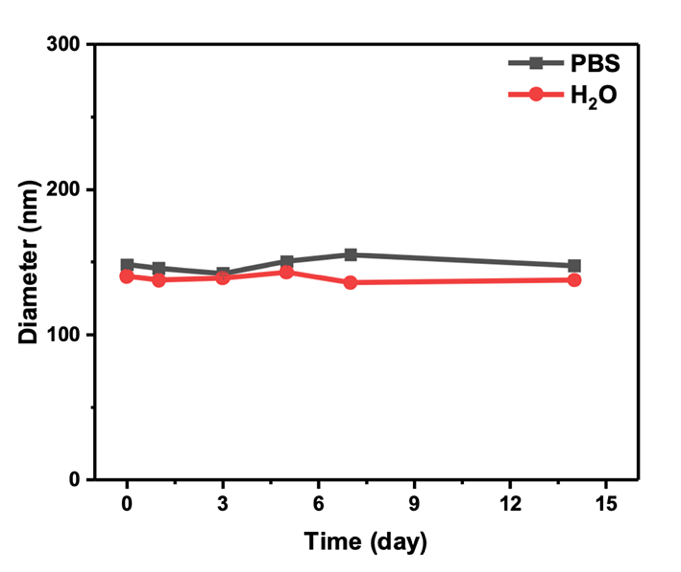


**Fig. S1.** The hydrodynamic diameter of FPP nanomicelles in PBS and water. (n=3)


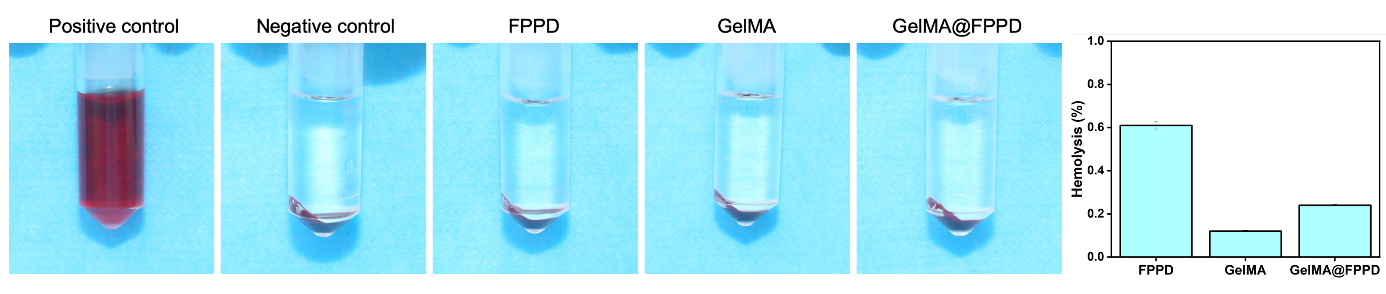


**Fig. S2.** Hemolytic activity assay. (n=3)


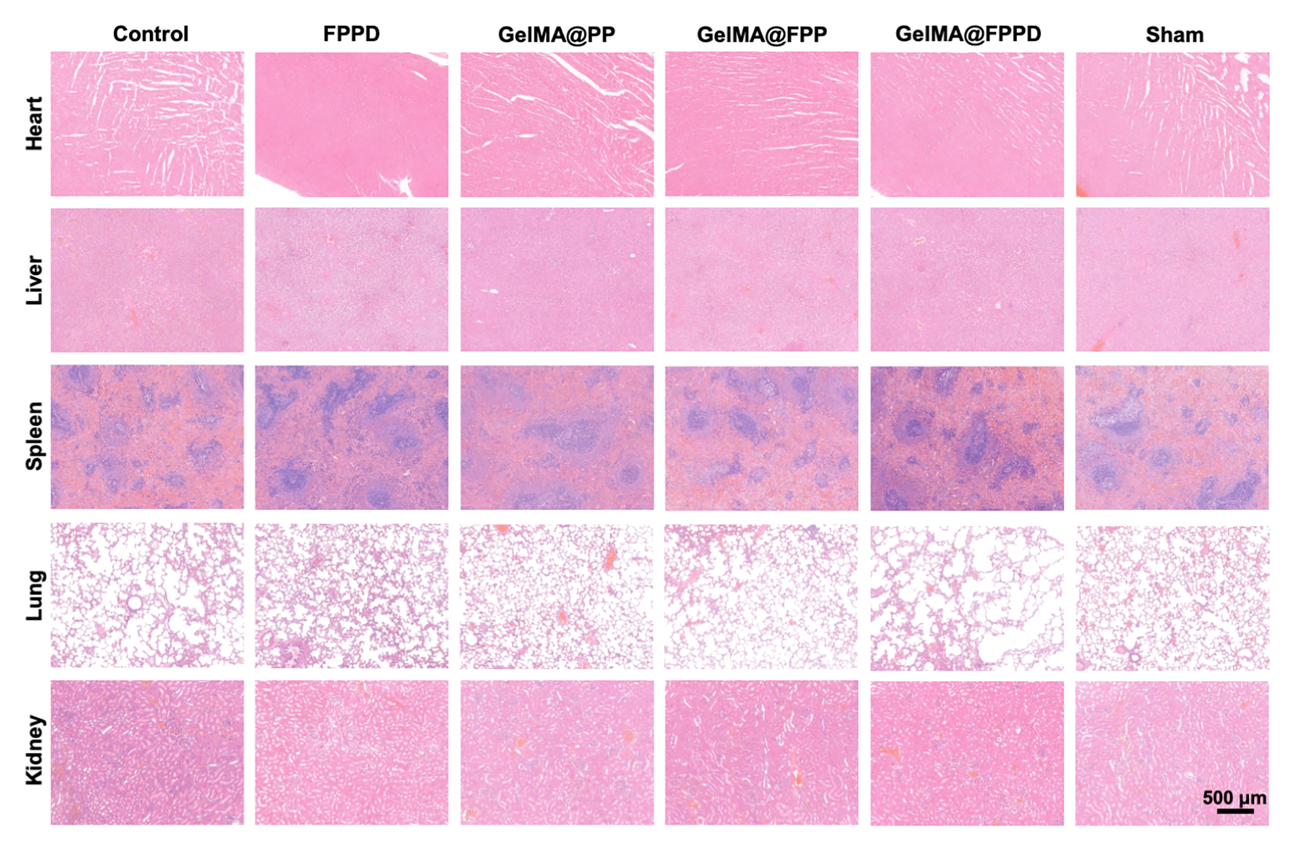


**Fig. S3.** H&E staining of vital systemic organs.

**Table S1.** Primer sequences of the genes for qRT-PCR analysis

| Gene | Forward primer (5’-3’) | Reverse primer (5’-3’) |
| --- | --- | --- |
| β-actin | GTGACGTTGACATCCGTAAAGA | GTAACAGTCCGCCTAGAAGCAC |
| TNF-α | GTTCCCAAATGGCCTCCC | GTGCTCCTCACCCACACCG |
| iNOS | GCCCAGGAGGAGAGAGAT | GCAAAGAGGACTGTGGCT |

**Reference**

1. Yang, J.; Zhu, Y.; Wang, F.; Deng, L.; Xu, X.; Cui, W., Microfluidic liposomes-anchored microgels as extended delivery platform for treatment of osteoarthritis. *Chem Eng J* **2020,** *400*, 126004.

2. Yang, J.; Han, Y.; Lin, J.; Zhu, Y.; Wang, F.; Deng, L.; Zhang, H.; Xu, X.; Cui, W., Ball-Bearing-Inspired Polyampholyte-Modified Microspheres as Bio-Lubricants Attenuate Osteoarthritis. *Small* **2020,** *16* (44), e2004519.

3. Yang, J.; Liang, J.; Zhu, Y.; Hu, M.; Deng, L.; Cui, W.; Xu, X., Fullerol-hydrogel microfluidic spheres for in situ redox regulation of stem cell fate and refractory bone healing. *Bioact Mater* **2021,** *6* (12), 4801-4815.

4. Li, X.; Wang, X.; Liu, Q.; Yan, J.; Pan, D.; Wang, L.; Xu, Y.; Wang, F.; Liu, Y.; Li, X.; Yang, M., ROS-Responsive Boronate-Stabilized Polyphenol-Poloxamer 188 Assembled Dexamethasone Nanodrug for Macrophage Repolarization in Osteoarthritis Treatment. *Adv Healthc Mater* **2021**, e2100883.

5. Slattery, C.; Kweon, C. Y., Classifications in Brief: Outerbridge Classification of Chondral Lesions. *Clin Orthop Relat Res* **2018,** *476* (10), 2101-2104.

6. Moon, S. J.; Woo, Y. J.; Jeong, J. H.; Park, M. K.; Oh, H. J.; Park, J. S.; Kim, E. K.; Cho, M. L.; Park, S. H.; Kim, H. Y.; Min, J. K., Rebamipide attenuates pain severity and cartilage degeneration in a rat model of osteoarthritis by downregulating oxidative damage and catabolic activity in chondrocytes. *Osteoarthr Cartilage* **2012,** *20* (11), 1426-38.
